# Malignant Superior Vena Cava Syndrome: State of the Art

**DOI:** 10.7759/cureus.20924

**Published:** 2022-01-04

**Authors:** Vasileios Patriarcheas, Maria Grammoustianou, Nikolaos Ptohis, Ioanna Thanou, Minas Kostis, Ioannis Gkiozos, Andriani Charpidou, Ioannis Trontzas, Nikolaos Syrigos, Elias Kotteas, Evangelos Dimakakos

**Affiliations:** 1 Internal Medicine, Thoracic Diseases General Hospital Sotiria, Athens, GRC; 2 Oncology, Thoracic Diseases General Hospital Sotiria, Athens, GRC; 3 Interventional Radiology, General Hospital of Athens G. Gennimatas, Athens, GRC; 4 Internal Medicine, General Hospital of Karpathos, Karpathos, GRC

**Keywords:** superior vena cava syndrome, superior vena cava obstruction, malignancy-related superior vena cava syndrome, thoracic oncology, non-hodgkin lymphoma (nhl), lung cancer, endovascular therapy

## Abstract

Superior vena cava syndrome (SVCS) is a clinical entity characterized by signs and symptoms arising from the obstruction or occlusion of the thin-walled superior vena cava (SVC) and can result in significant morbidity and mortality. Despite the rise of benign cases of SVCS, as a thrombotic complication of intravascular devices, it is most commonly seen secondary to malignancy as a consequence of thrombosis, direct invasion of tumor cells inside the vessel, or external compression. SVCS can be the initial presentation of a previously undiagnosed tumor in up to 60% of cases. Lung cancer and non-Hodgkin lymphoma (NHL) are responsible for up to 85%-90% of malignancy-related SVCS, while metastatic cancers account for approximately 10%. Herein, we review the pathophysiology, etiology, clinical presentation, diagnosis, and management of malignancy-related SVCS.

## Introduction and background

Superior vena cava syndrome (SVCS), which William Hunter first described in 1757 [1], encompasses a collection of signs and symptoms arising from the obstruction of blood flow through the thin-walled superior vena cava (SVC). Clinical symptoms include cough, dyspnea, and orthopnea, while facial edema and plethora, upper extremity swelling, and dilation of the chest wall and neck veins are the most commonly encountered signs [2]. Malignant and nonmalignant causes can provoke SVC obstruction. In malignancy-related SVCS, the obstruction can occur due to intraluminal tumor cell invasion, external compression by adjacent pathologic processes, or thrombosis [3]. Before antibiotics came into widespread clinical use, SVCS was usually of infectious origin, with aortic aneurysms due to tertiary syphilis and mediastinal adenopathy due to tuberculosis being common causes of SVCS. Nowadays, nonmalignant cases of SVCS are arising as a consequence of thrombus formation associated with intravascular devices such as catheters and pacemakers. Notwithstanding the fact that during the last decades, the overall incidence of benign cases of SVCS has risen and accounted for up to 40% of all cases, the majority of SVCS cases are the result of mediastinal malignancies, such as lung cancer (up to 10% of patients with small cell lung cancer (SCLC) and 2%-4% of all patients with lung cancer), non-Hodgkin lymphoma (NHL), and metastatic tumors [4,5]. SVC obstruction was traditionally considered a relative emergency requiring immediate intervention; it is now well established that patients with SVCS rarely experience life-threatening complications [6,7]. Accurate diagnosis and biopsy should precede any emergent therapeutic approach, considering that SVCS can be the initial presentation of a previously undiagnosed tumor in up to 60% of patients presenting with SVC obstruction symptoms [8].

## Review

Anatomy and physiology

The superior vena cava (SVC) is a thin-walled and low-pressure valveless vein that carries deoxygenated blood - approximately one-third of the venous return - from the head, neck, upper extremities, and torso to the heart [9]. The SVC is formed posterior to the inferior border of the first right costal cartilage by the union of the right and left brachiocephalic veins (i.e., innominate veins), which in turn are formed by the union of the internal jugular and subclavian veins. The azygos vein drains into the SVC via the azygos arch behind the first intercostal space before it pierces the pericardium. The SVC descends into the right side, coursing through the superior and middle mediastinum and flowing into the right atrium, at the level of the third intercostal space. The SVC is about 7 cm long and 2 cm wide, and its lower part is covered by the pericardium [9,10]. The SVC is susceptible to obstruction due to pathological processes in the adjacent, relatively rigid structures such as the sternum, trachea, pulmonary artery, right mainstem bronchus, and numerous lymph nodes, resulting in extrinsic compression. Apart from that, the SVC can be obstructed because of direct tumor invasion. As a consequence of SVC obstruction, the venous pressure in collateral vessels increases, forming an alternative network for the return of venous blood to the right atrium. This collateral blood flow network includes the azygos, hemiazygos, internal mammary, lateral thoracic, and vertebral pathways [11]. The azygos vein is formed by the union of the right subcostal and right ascending lumbar veins. It is a collateral venous pathway of great importance, as it serves as a connection between the superior and inferior vena cava [12]. Therefore, an obstruction of the SVC below the insertion of the azygous vein is less tolerated and result in more intense symptoms compared to a block above the orifice of the azygos, where blood is diverted to the heart via the inferior vena cava and azygous system [13]. Typically, the venous collaterals dilate over a few weeks to create an adequate network to handle the blood flow that normally drains through the SVC to the right atrium. Consequently, there is a notable elevation in the upper body venous pressure, which gradually decreases. Nevertheless, central venous pressures remain elevated even when a fully developed venous collateral network is present, giving rise to SVCS’s characteristic signs and symptoms.

Etiology of SVCS

As already mentioned, for several centuries, infectious diseases - especially syphilis and tuberculosis - were the leading causes of SVC obstruction. Due to the introduction of antibiotics into widespread clinical usage, intrathoracic malignancies have emerged as the most common cause of SVC obstruction (Table 1). More specifically, mediastinal malignancies are responsible for more than two-thirds of SVCS cases and can be the initial presentation of a previously undiagnosed tumor [14]. Lung cancer and non-Hodgkin lymphoma (NHL) are responsible for 85% of malignancy-related SVCS cases. Small cell lung cancer (SCLC) and squamous cell carcinoma are the two most frequent lung cancer histologic types associated with SVCS. SVC obstruction is more common in patients with SCLC; however, the higher incidence of non-small cell lung cancer (NSCLC) makes it the most widespread cause (≈50% of the cases) of malignancy-related SVCS [15]. Non-Hodgkin lymphoma (NHL) is the second most common malignant cause of SVC obstruction, being responsible for 10% of malignancy-related cases [16]. Metastatic cancers account for the rest (≈10%-15%) of SVCS cases [5,15]. Any tumor that can metastasize in the mediastinum can give rise to SVCS for mediastinal, but breast cancer is the most frequent primary tumor site in up to 70%. Germ cell cancer, thymoma, and mesothelioma represent rare causes of malignant SVC obstruction [17]. On the other hand, the widespread use of indwelling central venous catheters and implanted devices during the last decades has led to an increase in the incidence of nonmalignant (benign) causes of SVC obstruction. Thrombi associated with these catheters and parts of implanted devices (e.g., pacemaker leads) have emerged as a significant cause of SVCS and account for up to 30% of all cases [6,7]. Additional rare benign non-device-related causes of SVCS include mediastinal fibrosis, radiation fibrosis, retrosternal goiter, Bechet’s syndrome, and iatrogenic causes [18-20].

**Table 1:** Etiologies of superior vena cava syndrome [9,15]

Malignancy-related SVCS (~70%)	Benign SVCS (~30%)
Non-small cell lung cancer	Indwelling central venous catheters, implanted devices (e.g., pacemakers)
Small cell lung cancer	Mediastinal fibrosis, radiation fibrosis, retrosternal goiter, Bechet’s syndrome
Non-Hodgkin lymphoma	Iatrogenic causes
Metastatic and other tumors	

Clinical presentation and grading system

The clinical manifestations of SVCS are determined by increased venous pressure in the upper body due to SVC obstruction. The presentation of SVCS varies, depending on the anatomical level, rapidity, the degree of SVC obstruction, and the extent of the venous collateral network [9]. In addition, the time of onset of symptoms varies as well, but typically, SVCS develops over weeks to months. Independently from the cause of SVC obstruction, face or neck swelling accompanied by dyspnea are common presenting symptoms, and patients frequently complain of a sense of head “fullness.” Other common signs and symptoms include cough, distended chest and neck veins, arm edema, and dizziness aggravating by bending forward [7,9,15]. Less frequent signs and symptoms include upper body cyanosis, hoarseness, stridor, and neurological manifestations such as headache, vision changes, confusion, or even syncope [10]. The later signs and symptoms are associated with more severe cases of SVCS. A grading system has been proposed by Yu et al., which stratifies the severity of SVCS symptoms to determine the urgency of intervention and facilitates communication between physicians (Table 2) [21]. This grading system assesses the degree of edema (laryngeal and/or cerebral) and hemodynamic status to categorize SVCS between life-threatening (grade 4), severe (grade 3), and non-life-threatening cases (grade 0-2).

**Table 2:** Grading system for superior vena cava syndrome [21]

Grade	Findings	Estimated incidence (%)
0	Asymptomatic – radiographic superior vena cava obstruction in the absence of symptoms	10
1	Mild – edema in the head or neck (vascular distention), cyanosis, plethora	25
2	Moderate – edema in the head or neck with functional impairment (mild dysphagia; cough; mild or moderate impairment of the head, jaw, or eyelid movements; visual disturbances caused by ocular edema)	50
3	Severe – mild or moderate cerebral edema (headache, dizziness), mild/moderate laryngeal edema, or diminished cardiac reserve (syncope after bending)	10
4	Life-threatening – significant cerebral edema (confusion, obtundation), significant laryngeal edema (stridor), or significant hemodynamic compromise (syncope without precipitating factors, hypotension, renal insufficiency)	5
5	Fatal – death	<1

The life expectancy of patients with malignant SVCS syndrome is approximately six months but varies widely depending on the underlying neoplasm, which is an important prognostic parameter. According to a study by Holliday et al., it seems that a lower grade of SVCS is associated with an increased likelihood of discharge after treatment [22].

Diagnosis

**Figure 1:**
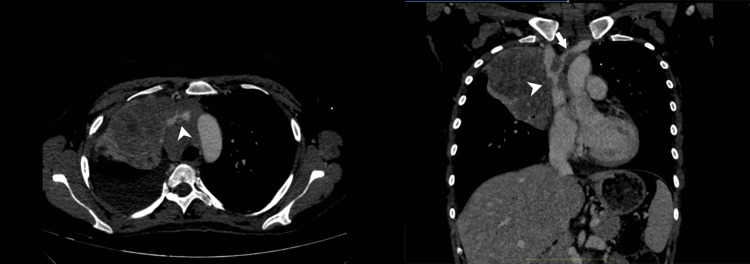
CT axial and coronal images of a patient with diffuse large B-cell lymphoma causing severe mass effect on SVC and right brachiocephalic vein

Diagnosis of SVCS can be suspected in a patient who presents with a combination of the aforementioned signs and symptoms. Therefore, patient evaluation has to commence with a detailed medical history and physical examination, focusing on the characteristic symptoms and signs of SVCS. Confirmation of a diagnosis of SVC obstruction requires imaging (Figure 1). Imaging modalities include chest radiography (CXR), contrast-enhanced computed tomography (CECT), duplex ultrasound, conventional venography, and magnetic resonance venography. Imaging plays a crucial role since it can differentiate between mediastinal masses or intraluminal thrombi as causes of SVCS and helps choose the best treatment option. Chest radiography (CXR) can show indirect signs such as mediastinal widening, pleural effusion, lung masses, and right hilar lymphadenopathy. Contrast-enhanced computed tomography (CECT) is the most helpful imaging modality since it can define the extent of venous blockage, illustrate collateral venous pathways, and discriminate the cause of SVC obstruction (i.e., extrinsic compression versus thrombosis) [23]. In addition, the presence of collateral vessels in CECT is strongly associated with SVC obstruction (sensitivity, 92%; specificity, 96%) [24]. Moreover, CECT can reveal the adjacent pathologic processes such as mediastinal masses, lung masses, and lymphadenopathy. Despite the fact that SVC cannot be directly visualized using ultrasound due to the acoustic shadow of the overlying ribs, the duplex ultrasound of the upper extremities is useful for detecting thrombus in subclavian axillary and brachiocephalic veins, especially in catheter-related thrombosis. Moreover, it is used for ultrasound-guided IV access for endovascular therapy and venography [9,23]. Conventional catheter-based central venography is the gold standard for the identification of SVC obstruction, collateral venous pathways, and the extent of associated thrombus formation [24]. Apart from that, conventional venography provides the opportunity to intervene therapeutically for revascularization. Magnetic resonance venography constitutes an alternative approach in patients with an allergy to iodinated contrast media used in CECT or for patients in whom venous access cannot be obtained for conventional catheter-based venography [23]. Aside from imaging and because of the fact that SVC obstruction can be the presenting symptom of a previously undiagnosed tumor, accurate histologic diagnosis is crucial to confirm malignancy. Tissue sampling strategies might include either an accessible peripheral biopsy site (e.g., a lymph node or a peripheral lung nodule) or more invasive procedures such as bronchoscopy or bone marrow (BM) aspiration in the case of a hematologic malignancy [10].

Treatment options

Evidence-based guidelines for the management of SVCS are not available. Most of the options regarding the therapeutic approach of SVC obstruction are obtained from case series and randomized trials. Management of SVCS should be multidisciplinary with cooperation among different medical specialties such as radiology and interventional radiology, pulmonology, surgery, vascular surgery, and oncology. The therapeutic approach of SVCS has two pillars: to alleviate symptoms related to SVC obstruction and to treat the underlying disease. Treatment approaches for SVC obstruction include radiation therapy, chemotherapy, open surgery, and endovenous recanalization. During the past decades, SVCS was considered a medical emergency. Nowadays, it is well known that SVC obstruction rarely manifests as a life-threatening entity due to laryngeal or cerebral edema. In these cases, initial stabilization of airway, breathing, and circulation (ABC) and urgent recanalization are sine qua non [5,25,26].

General Measures

Regardless of the etiology, head elevation should be the initial mainstay management in all patients because it decreases head and neck edema and hydrostatic pressure [9]. Other general measures include the administration of glucocorticoids and loop diuretics. Glucocorticoids can be effective in two settings: in steroid-sensitive malignancies such as lymphoma and thymoma, where they can reduce tumor burden and SVC obstruction, and as prophylaxis for radiation-induced edema in order to minimize airway obstruction, especially in patients with preexisting laryngeal edema [10]. Loop diuretic administration has been recommended in the literature - without clear evidence of efficacy - as part of the initial approach to SVCS because diuresis reduces venous return to the heart and relieves the increased pressure [17]. However, this approach has begun to receive strong criticism because the problem is not the fluid overload but the obstruction of blood flow through the SVC.

Radiation Therapy

Radiation therapy (RT) was the gold standard method and the fastest way to relieve obstruction in patients with life-threatening malignancy-related SVCS. This was due to the belief that lung cancer, which is radiosensitive, would be the most probable cause of SVC obstruction and encompasses a true medical emergency; thus, any diagnostic procedure such as tissue sampling before RT will cause delayed management that can turn fatal [27]. Nevertheless, RT is no longer considered the best option. First of all, endovenous recanalization has a faster way of alleviating symptoms (usually within 0-72 hours) compared with RT, where it can take up to two weeks to improve [28]. Moreover, RT has an efficacy rate of up to 80%; thus, 20% of patients do not experience symptomatic relief [29]. Finally, a post-RT biopsy is sometimes associated with loss of pathologic diagnosis in up to 42% of cases [30]. Therefore, for all the above reasons, endovascular therapy is superior to RT.

Chemotherapy

In patients with chemotherapy-sensitive malignancies such as SCLC, lymphoma, and germ cell tumors who present with non-life-threatening manifestations, systemic chemotherapy can effectively palliate the symptoms of SVCS obstruction. The results of chemotherapy alone are usually seen within one to two weeks of treatment initiation and can achieve complete relief of symptoms of SVCS in up to 80% of patients with non-Hodgkin lymphoma and SCLC. If chemotherapy is the initial treatment modality and SVC obstruction is unresolved, it should be administered through veins in the lower extremity [5,17,31].

Surgery

Open surgical intervention such as bypass grafting procedure plays a limited role in SVCS management. However, in some SVCS cases, open surgery can still be the best treatment approach in selected patients with an impact on prognosis. Malignant invasion is the most frequent indication for SVC resection and reconstruction. For example, advanced thymic neoplasms can cause SVC obstruction by protruding intraluminally into the left brachiocephalic vein and SVC. In these patients, to achieve microscopically margin-negative resection (R0), which is essential for prolonged survival, SVC resection and reconstruction are indicated. In addition, for the same reason, surgical intervention could be considered a therapeutic approach in patients with germ cell tumors [31].

Endovascular Therapy

**Figure 2:**
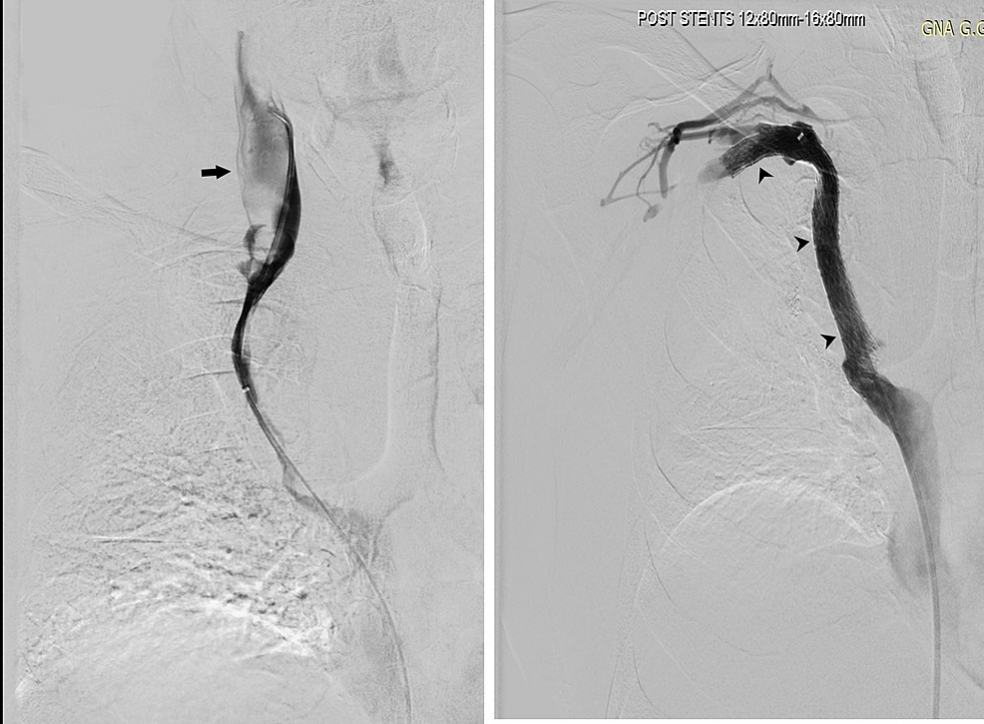
Venography depicting extensive stenosis of the SVC together with thrombosis (left) and recanalization of the lesion with combined thromboaspiration and stent placement technique (right)

Endovascular therapy (ET) should be considered the standard of care approach in patients with malignancy-related symptomatic SVCS and short to medium life expectancy either at initial presentation or following RT or chemotherapy. Since the first SVCS stenting by Charnsangavej et al. in 1986, endovascular therapy has emerged as the standard of care approach in SVC obstruction. ET offers many advantages, including a high technical success rate (95%-100%), high efficacy rate (over 90% of patients report relief of symptoms), and low complication rate. Moreover, ET results are permanent in up to 90% with an average recurrence rate of 10.5% (ranging from 1.2% to 20.5%), and ET can be combined with other treatment options, i.e., radiotherapy and chemotherapy [9,32,33]. Venous access is typically obtained under ultrasound guidance through the internal jugular, basilic/brachial, or femoral veins. Superior vena cava cavography precedes stent placement in order to confirm the extent of SVC obstruction and the presence or absence of associated thrombus. Stenting is usually performed in the supine position using conscious sedation and local anesthesia. Usually, an intravenous bolus of heparin (3,000-5,000 IU) is administered prior to stent deployment; however, this practice is not universal. Occasionally, it may be necessary to perform standard balloon angioplasty (predilation) to facilitate stent delivery and deployment [25]. Many factors can influence the type of stent selected, such as severity, length, and resistance to dilation. Thus, the interventionalist can choose between a variety of available stents, including balloon-expandable, self-expanding, and self-expanding nitinol stent coated with polytetrafluoroethylene and stent grafts [9]. If occlusion extends to both brachiocephalic veins, “kissing stents” (Y-shaped) in addition to a separate SVC stent can be an option for the interventionalist, although alleviation of the obstruction in one of the blocked brachiocephalic vein is often sufficient and bilateral stenting is associated with higher rates of complications [34]. Complications of SVC stenting - periprocedural and postprocedural - are uncommon, with a reported incidence of less than 8%. Complications can be less severe, including access site infection and hematoma formation, or even life-threatening, such as pericardial tamponade, pulmonary embolism, SVC rupture, and stent migration. The overall procedure-related mortality is 2% [8,9]. In some cases of SVC obstruction, there is a superimposed thrombosis (Figure 2). Generally, clot-related SVC obstruction occurs more frequently due to benign causes; however, malignancy as a hypercoagulable state can lead to thrombus formation in the SVC. These patients are usually symptomatic and have a high Khorana score [35-37]. In these cases, catheter-directed thrombolysis (CDT) or aspiration thrombectomy is recommended prior to recanalization in order to avoid pulmonary embolism. Thrombus removal alleviates symptoms of SVC obstruction and reveals the length of the lesion that has to be treated [33]. Thrombolytic agents increase the risk of bleeding, and if absolute contraindications to thrombolysis are present, mechanical thrombectomy may be preferred [36]. After thrombolysis or thrombectomy, cavography is repeated in order to evaluate SVC patency, and in the absence of a thrombus, the interventionalist can proceed to stent placement.

Long-Term Antithrombotic Treatment

Published studies regarding post-reconstruction anticoagulation therapy after endovascular recanalization in the absence of significant thrombosis are insufficient, and current therapeutic practices vary. There is disunity on the approach for preventing stent reocclusion in malignancy-related SVC obstruction, with some experts supporting anticoagulation for at least three months [38,39] and others lifelong [40,41], while others advocate antiplatelet therapy alone [42]. Because no evidence-based guidelines exist, weighing the risk and benefits of anticoagulation is essential. In patients with superimposed thrombosis, full anticoagulation with LMWH or DOACS after thrombus removal is indicated for three to six months [8].

## Conclusions

Superior vena cava obstruction is most commonly related to an underlying malignancy and can result in significant morbidity and mortality. SVCS does not represent a true oncologic emergency in the vast majority of cases; however, it necessitates expedient management and prompt investigation for occult malignancy in patients who present with unknown history of cancer. SVCS does not affect the chance of cure of the underlying malignancy and should not change the overall treatment. Management involves symptom alleviation and treatment of underlying disease and has evolved over time. In the past, radiation therapy (RT) was considered the first-line treatment and the quickest way to relieve symptoms of SVC obstruction. Nowadays, endovascular therapy (ET) is considered the standard of care approach, providing rapid relief with high efficacy, without adversely affecting subsequent treatment with radiotherapy or chemotherapy when it is needed. Unfortunately, evidence-based guidelines for the management of SVCS are lacking, and in the years to come, there is a need for the establishment of therapeutic strategies in order to warrant the best treatment approach in this old clinical problem.
